# Transcriptome analysis of Harumi tangor fruits: Insights into interstock-mediated fruit quality

**DOI:** 10.3389/fpls.2022.995913

**Published:** 2022-10-13

**Authors:** Ling Liao, Yunjie Li, Xiaoyi Bi, Bo Xiong, Xun Wang, Honghong Deng, Mingfei Zhang, Guochao Sun, Zhenghua Jin, Zehao Huang, Zhihui Wang

**Affiliations:** ^1^ College of Horticulture, Sichuan Agricultural University, Chengdu, China; ^2^ Institute of Pomology and Olericulture, Sichuan Agricultural University, Chengdu, China

**Keywords:** interstock, rootstock, fruit quality, genetic factor, development, harumi tangor

## Abstract

Harumi tangor fruit with Ponkan as an interstock contains significantly higher levels of total soluble solids compared to Harumi tangor fruit cv.with no interstock. Transcriptome analysis of two graft combinations (Harumi/Hongjv (HP) and cv. cv.Harumi/Ponkan/Hongjv (HPP)) was conducted to identify the genes related to use of the Ponkan interstock. Soluble sugars and organic acids were also measured in the two graft combinations. The results showed that the contents of sucrose, glucose, and fructose were higher in the fruits of HPP than in those of HP; additionally, the titratable acid levels were lower in grafts with interstocks than in grafts without interstocks. Transcriptome analysis of HPP and HP citrus revealed that the interstock regulated auxin and ethylene signals, sugar and energy metabolism, and cell wall metabolism. Trend and Venn analyses suggested that genes related to carbohydrate-, energy-, and hormone-metabolic activities were more abundant in HPP plants than in HP plants during different periods. Moreover, weighted gene co-expression network analysis demonstrated that carbohydrates, hormones, cell wall, and transcription factors may be critical for interstock-mediated citrus fruit development and ripening. The contents of ethylene, auxin, cytokinin, transcription factors, starch, sucrose, glucose, fructose, and total sugar in HPP plants differed considerably than those in HP fruits. Interstocks may help to regulate the early ripening and quality of citrus fruit through the above-mentioned pathways. These findings provide information on the effects of interstock on plant growth and development.

## Introduction

Citrus fruits represent one of the most important crops worldwide (Conti et al., 2021) and are grown predominantly in tropical and subtropical areas. Citrus fruits possess abundant primary metabolites, such as organic acids, sugars, and amino acids. Moreover, they possess various secondary metabolites and volatile compounds that contribute to their nutritional characteristics and exert health benefits (Batista-Silva et al., 2018). Organic acid is the key factor affecting the fruit sensory quality (Fan et al., 2017), The acid content of fruit is determined by the balance of acid synthesis and degradation. Genes regulate changes in organic acids during fruit development (Zhang et al., 2021). For example, Lin et al. (2022) found that *PBWRKY10* promotes the accumulation of organic acids in pears. The sugar-to-acid ratio is also an important determinant of fruit maturity (Jiang et al., 2018). Sucrose is transported into fruit *via* the phloem as part of the sugar metabolic pathway, after which neutral invertase converts sucrose into glucose and fructose or sucrose synthase converts sucrose into UDP-glucose and fructose (Umer et al., 2020). However, citrus trees have long juvenile periods, which often span more than 6 years, before reaching full maturity (Cuenca et al., 2018).

Grafting is a well-developed technique used for fruit trees in which a scion and rootstock are combined to form a new plant with a blend of each plant’s characteristics, and is the most important reproduction mode for citrus production (Wu et al., 2019). Many studies have demonstrated that grafting can alter gene expression in scions (Shen et al., 2019) and is an effective method for shortening the juvenile period (Habibi et al., 2022). Rootstock selection is crucial for commercial cultivation of citrus fruits, as it affects the fruit yield and quality (Santos et al., 2017), including the juice total soluble solid (TSS) and acidity levels, vitamin C content, and antioxidant capacity (Carolina et al., 2019). Moreover, rootstocks improve resistance to biotic and abiotic stresses and affect the plant water status and photosynthesis (Albrecht et al., 2020). The mechanism triggering these effects likely involves differences in hormonal signaling, gene expression, metabolites, and ion uptake and transport in grafted trees (Goldschmidt, 2014). Auxin was the first discovered plant hormone and plays a key role in regulating fruit ripening (Kou et al., 2022). In a study of graft compatibility in citrus trees (He et al., 2018), auxin was shown to cooperate with several hormones during graft union formation (Nanda and Melnyk, 2017). Ethylene is also critical for controlling the ripening of climacteric and non-climacteric fruits (Xu et al., 2018). Gibberellins play major roles in the network of floral-induction pathways and in regulating female flower differentiation (Khan et al., 2014). Abscisic acid may be a key factor in non-climacteric fruit ripening (Li et al., 2022). In plants, phytohormones do not function independently, and fruit ripening is governed by interactions between phytohormones (Fenn and Giovannoni, 2021).

To adjust the variety structures of fruit trees, high grafting can be performed to eliminate inferior varieties by generating ‘interstocks’ (Gil-Izquierdo et al., 2004). Interstock grafting involves the use of different genetic material between the rootstock and selected commercial cultivar, with the resulting plant formed from three different individuals through a double-graft union (Calderón et al., 2021). Interstocks were initially studied as a tool for overcoming the interspecific incompatibility of different scion/rootstock combinations of potential agronomic interest (Rogers and Beakbane, 2003). More recently, studies have focused on interstocks of plum (Popara et al., 2020), apple (Zhou et al., 2021), peach (Calderón et al., 2021), and pear (Cui et al., 2021) to control scion vigor. Moreover, interstocks have been widely used in high-density planting systems by altering several important factors such as the tree structure, carbon distribution, and shoot-growth rate and duration (Zhou et al., 2015).

The mechanisms underlying interstock–scion interactions remain largely unknown. It is currently thought that the dwarfing mechanisms of rootstocks and interstocks alter the scion size, in which phytohormones may play important roles (Li et al., 2012). Heterografting upregulates the expression of genes involved in stress responses at the graft interface compared with the corresponding levels in autografted control citrus trees (Cookson et al., 2014). In addition, lemon trees grafted with interstocks show increased longevity, lemon production, and quality (Gil-Izquierdo et al., 2004). However, reports on interstock-mediated expression modifications in late-ripening citrus fruits are lacking. ‘Harumi’ (*Citrus reticulata* × *Citrus sinensis*) is a late-ripening cultivar that produces an oblate-shaped fruit; the rind color is orange and the fruit is easy to peel. The fruit ripens in January (Takishita et al., 2021). This cultivar has been increasingly planted in China and is favored by many consumers because of its taste, high yield, and abundant nutrients (Dong et al., 2020). Because interstocks greatly influence citrus quality and consumers have increasing demanded high-quality fruits, it necessary to carefully select interstocks that produce high-quality fruits. We hypothesized that when Hongjv (*C. reticulata* × *C. sinensis*) is used as the base rootstock, using Ponkan (*C. reticulata* Ponkan) as an interstock can improve the fruit quality of ‘Harumi’ tangor. Thus, the main objective of this study was to explore how interstocks improve the quality of a common ‘Harumi’ tangor fruit. This effect was investigated using common ‘Harumi’ tangor grafted onto Hongjv (HP, non-interstocks) and cv.Ponkan/Hongjv (HPP, with interstocks).

## Material and methods

### Plant materials and growth conditions

This study was performed at the Citrus Modern Agricultural Park in Qingshen (29°86′–29°91′ N and 103°90′–106°38′ E) in Sichuan, China. The park has an elevation of 133 m, an annual accumulated temperature of >3,400°C, 3,000 h of sunshine, an annual frost-free period of 172–188 days, and a mean annual precipitation of 693 mm. Eighteen 2-year-old Hongjv seedlings were planted in September 2016. The first grafting (rootstock grafted with interstock) was conducted in early April 2017; Nine Hongjv were chip-budded with Ponkan at a height of 10 cm above the ground to form a Ponkan/Hongjv combination. Nine controls without grafting were also planted. The second branch grafting was conducted in March 2018, the interstocks were grafted with cultivar ‘Harumi’ tangor cv. cv.at 5 cm from the interstock/rootstock union to form a Harumi/Ponkan/red orange combination (HPP). Plants without interstock were also grafted with ‘Harumi’ tangor to form a Harumi/red orange combination (HP) at 10cm from the soil level. Plants were allowed to grow with full nutrition and standard treatments for citrus from 2018 to 2022.

Fruit samples of ‘Harumi’ tangor were harvested at 45, 90, 135, and 180 days after flowering (DAF) from 2021 to 2022. Three biological replicates (three trees per replicate) were harvested at each developmental stage. The samples from the four stages were named as HP1, HP2, HP3, and HP4 for HP and HPP1, HPP2, HPP3, and HPP4 for HPP. Twenty-seven representative fruits (three fruits per tree) of each combination were sampled at each developmental stage. Fruits were transported to the laboratory on the day of harvest and then were divided into two groups. One group was used 15 samples for physiological index assessment, after measurement of TSS, single fruit weight, vertical diameter, and transverse diameter index, fruit flesh without skin from same batch of fruit were frozen in liquid nitrogen and stored at −80°C until high-performance liquid chromatography (HPLC) analysis. From the second group, the peel and flesh were isolated, and the flesh was frozen immediately in liquid nitrogen and stored at −80°C for subsequent analyses [i.e., RNA sequencing (RNA-seq) and quantitative real- time (qRT)-PCR].

### Physiological index assessment

A refractometer (HC-112ATC, Shanghai Lichenkeyi, China) was used for TSS measurements. The weight of each fruit was recorded using an electronic balance. The vertical and transverse diameters were measured for each fruit using a digital caliper gauge ( ± 0.01 mm).

### Soluble sugar and organic acid determinations

Soluble sugars and organic acids were extracted as described by Liao et al. (2019), with some modifications. Briefly, 2 g samples of frozen pulp were ground into a powder in liquid nitrogen, homogenized in 5.0 mL of ethanol (80%), and placed in a water bath at 80°C for 15 min. The homogenates were centrifuged at 9,000 ×*g* for 10 min at 4°C. The residues from each treatment were extracted in triplicate, and the supernatant of each extracted residue was collected into a 10 mL volumetric flask and brought to a final volume (10 mL) with 80% ethanol. The samples were filtered through a membrane filter (0.45 μm pore size) before injection into the HPLC system. The filtered solution was used for sugar and organic acid analyses.

The sugar content was analyzed using **HPLC** equipped with a refractive index detector (LC-1260; Agilent Technologies, Santa Clara, CA, USA). Samples were isolated on an Innoval NH_2_ column (4.6 × 250.0 mm, 5.0 μm; Bonna Agela, Wilmington, DE, USA). Acetonitrile: water = 80:20 (v:v) was used as the mobile phase for isocratic elution with the following parameters: sample volume, 10 µL; flow rate, 1.0 mL·min^-1^; column temperature, 30°C; detection temperature, 40°C.

Organic acids were analyzed using HPLC equipped with a UV detector (LC-1260; Agilent Technologies). Samples were isolated on a C18-WP column (4.6 × 250 mm, 5 µm, CNW Technologies, Shanghai, China). HPLC was performed using isocratic elution with the mobile phase (4% methanol solution; metaphosphoric acid was used to adjust the pH to 2.6). The parameters were as follows: sample volume, 10 µL; flow rate, 0.8 mL min^−1^; column temperature, 25°C; detection temperature, 25°C. Organic acids were detected at a wavelength of 210 nm. For the determination of sugars and organic acids, three biological replicates were taken and technical replications were performed three times.

### RNA-sequencing and data analysis

Eight samples of the pulp tissue of HP and HPP across four developmental stages were harvested, and three biological replicates were harvested for each sample. A total of 24 transcriptome profiles were obtained by RNA-seq using the Illumina HiSeq™ 4000 sequencing platform at Novogene (Beijing, China) (Liu et al., 2021).

The raw sequencing data were deposited in the National Center for Biotechnology Information (NCBI) Gene Expression Omnibus database under accession number PRJNA857756. The clean reads were aligned to the citrus reference genome (http://cucurbitgenomics.org/organism/21) using TopHat software version 2.0.12. Feature counting (genes, in this case) was performed using HTSeq software version 0.6.1. Gene abundances were calculated and normalized to the number of reads per kilobase per million reads according to Mortazavi et al. (2008), and differentially expressed genes (DEGs) between HP and HPP were identified using the DESeq R package. *P*-values < 0.05 were considered to reflect statistically significant differences and were adjusted using Benjamini and Hochberg correction (1995). We used the cluster Profiler package in R software to test for significant enrichment of the DEGs in Kyoto Encyclopedia of Genes and Genomes (KEGG) pathway analysis.

Total RNA was extracted from ‘Harumi’ tangor fruit pulp tissue using RNAprep Pure (Tiangen, Beijing, China), and first-strand cDNA was synthesized from total RNA using an RNA reverse transcription kit (Toyobo, Osaka, Japan) according to the manufacturer’s instructions. To verify the accuracy of the transcriptome data, we used Primer 5 to design specific primers and performed quantitative reverse transcription-PCR analysis (**Supplementary Table 1**). These primers were synthesized by Tsingke Biotech (Beijing, China). Quantitative reverse transcription-PCR analysis was performed using Bio-Rad CFX Manager (Hercules, CA, USA) and SYBR Premix Ex Taq II (novoprotein, Shanghai, China) to validate the DEG expression results. The citrus reference gene β-actin (GenBank: XM_006429010.2) was selected as an internal reference gene. The 2^–ΔΔCt^ method was used to analyze mRNA expression levels.

### Weighted gene co-expression network analysis and visualization

Co-expression and module analyses were performed using the R software package WGCNA 1.63 to study gene expression and trait variations (Langfelder and Horvath, 2008). WGCNA was conducted to construct a co-expression network including the TSS, single-fruit weight, sugar, and acid components; modules of highly correlated genes were identified using normalized expression matrix data. Genes with low fragments per kilobase of transcript per million mapped reads (FPKM) values (average FPKM <0.1) and a low coefficient of variation (<0.5) were excluded. A soft power threshold of 4 was interpreted as a soft threshold for the correlation matrix. The minimum module size was set to 30, and the minimum height of the merging modules was set to 0.25. Genes were clustered hierarchically according to the topological-overlap matrix measure.

### Statistical analysis

All statistical analyses were performed using SPSS software (version 19.0, SPSS, Inc., Chicago, IL, USA). One-way analysis of variance followed by Duncan’s multiple-range test was employed, and the standard deviation was calculated from three replicates. The differences between individual means were considered significant at *P* < 0.05.

## Results

### Variations among soluble sugars and organic acids during citrus fruit flesh development

**Figure 1:**
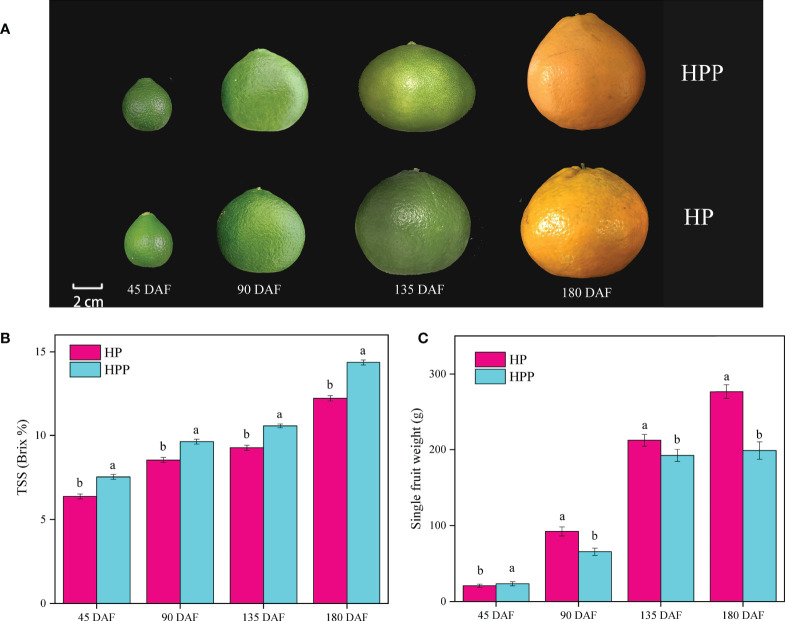
Changes in fruit phenotype, total soluble solid (TSS), and single-fruit weight between Harumi/Hongjv (HP) and Harumi/Ponkan/Hongjv (HPP). **(A)** Phenotypes, **(B)** TSS, and **(C)** single-fruit weights of HP and HPP fruits at 45, 90, 135, and 180 days after flowering (DAF). Each value shown represents the mean ± SD of three biological replicates.

Fruits of ‘Harumi’ tangor mandarins grown on HP and HPP trees were sampled at 45, 90, 135, and 180 DAF to explore the fruit quality at different stages of development (**Figure 1A**). TSS measured at 45, 90, 135, and 180 DAF (**Figure 1B**) showed significant variations. The TSS increased from 6.37 and 7.53 Brix% at 45 DAF to 12.23 and 14.37 Brix% at 180 DAF in HP and HPP, respectively. Differences in single-fruit weights were observed at different developmental stages (**Figure 1C**). For example, HP had mean single-fruit weights of 20.77, 92.18, 212.12, and 276.65 g at 45, 90, 135, and 180 DAF, respectively, whereas those of HPP were 23.33, 65.48, 192.20, and 198.86 g, respectively.

**Figure 2:**
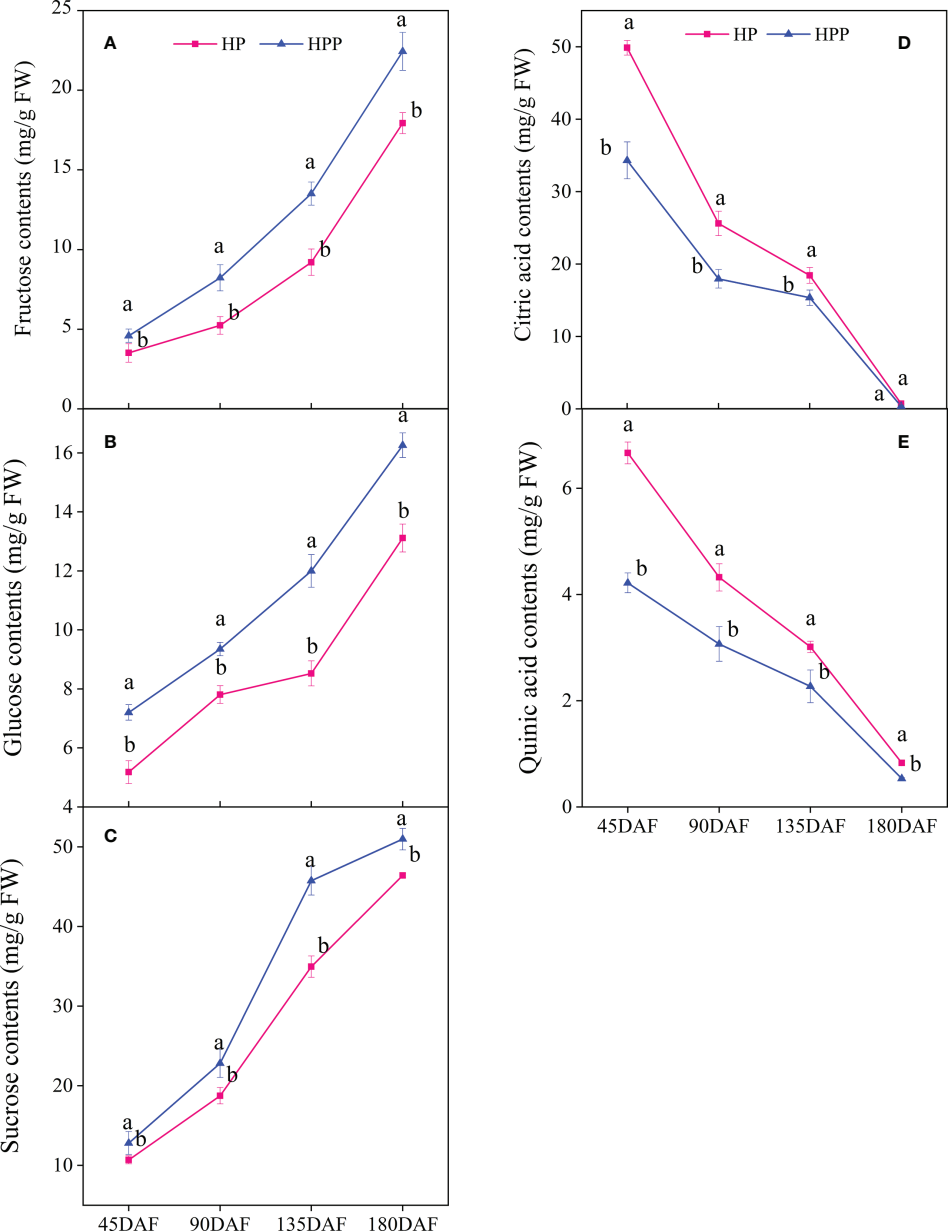
Changes in sugar and acid content between Harumi/Hongjv (HP) and Harumi/Ponkan/Hongjv (HPP). **(A)** fructose contents, **(B)** glucose contents, **(C)** sucrose contents, **(D)** citric acid contents, **(E)** quinic acid contents in HP and HPP fruits at 45, 90, 135, and 180 days after flowering (DAF). Each value shown represents the mean of three biological replicates.

The contents of soluble sugar (i.e., sucrose, glucose, and fructose) significantly differed during citrus fruit development (**Figures 2A–C**). The sucrose contents were 10.70, 18.76, 34.95, and 46.41 mg/g fresh weight (FW) at 45, 90, 135, and 180 DAF in HP, respectively, whereas those in HPP were 12.81, 22.82, 45.74, and 50.97 mg/g FW, respectively. The same trend was observed for fructose at all stages of citrus fruit development, i.e., 3.51,5.24, 9.21, and 17.93 mg/g FW and 4.59, 8.23, 13.51, and 22.44 mg/g FW at 45, 90, 135, and 180 DAF in HP and HPP, respectively. The glucose content was low at 45 DAF (5.18 and 7.20 mg/g FW) but increased significantly at 90 DAF (7.81 and 9.35 mg/g FW) and 135 DAF (8.53 and 12.00 mg/g FW) and 180 DAF (13.12 and 16.26 mg/g FW) in HP and HPP fruits, respectively. In this study, citric acid was the major acid detected in both sample types. The citric acid content decreased from 49.87 and 34.32 mg/g FW at 45 DAF to 0.72 and 0.36 mg/g FW at 180 DAF in the HP and HPP samples, respectively (**Figure 2D**). The same trend was observed for quinic acid at all stages of citrus fruit development, where the concentration was higher at 45 DAF in HP and HPP samples but decreased as the fruits neared maturity, i.e., to 0.83 mg/g FW in HP and 0.53 mg/g FW in HPP (**Figure 2E**).

### Transcriptome analysis of HP and HPP fruits

The HP and HPP pulp was harvested at 45, 90, 135, and 180 days after bloom (**Figure 1A**) and the transcriptomes were sequenced to construct 24 cDNA libraries. A total of 161.31 Gb of clean data were filtered, and 6 Gb of clean data were obtained for each sample, with a Q30 base percentage of >92%. When the sequencing data were aligned to the reference genome, >87.03% of clean reads were mapped. Less than 3% of reads could not be mapped (**Supplementary Table 2**).

**Figure 3:**
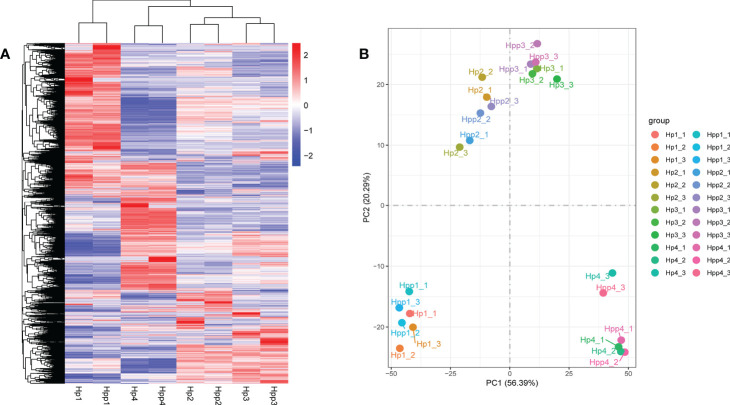
Clustering and Principal Component Analysis of Differential Metabolites between Harumi/Hongjv (HP) and Harumi/Ponkan/Hongjv (HPP) fruits. **(A)** Cluster heat map of differentially expressed genes (DEGs) between different samples, where red represents upregulation and blue represents downregulation. **(B)** Principal component analysis of the Harumi/Hongjv (HP) and Harumi/Ponkan/Hongjv (HPP) groups at 45, 90, 135, and 180 days after flowering (DAF). HP1, HP2, HP3 and HP4 represent HP at 45, 90, 135, and 180 DAF, respectively; and HPP1, HPP2, HPP3 and HPP4 represent HPP at 45, 90, 135, and 180 DAF, respectively. Each value shown represents the mean of three biological replicates.

The gene-expression levels (FPKM values) of fruit pulps harvested after 45 days were high in both the HP and HPP samples (**Supplementary Figure 1A**). Pearson’s correlation coefficient between the samples ranged from 0.575–0.987 (**Supplementary Figure 1B**). Differential expressed genes were displayed in a clustered heat map (**Figure 3A**), and principal component analysis showed that the treatment replicates clustered together, indicating the reliability of the sampling (**Figure 3B**). The first two components, which explained 61% of the variation, distinguished the four different treatment groups, with three biological replicates clustered together; each group showed discernable differences in their gene-expression profiles.

### DEGs in HP and HPP fruits

**Figure 4:**
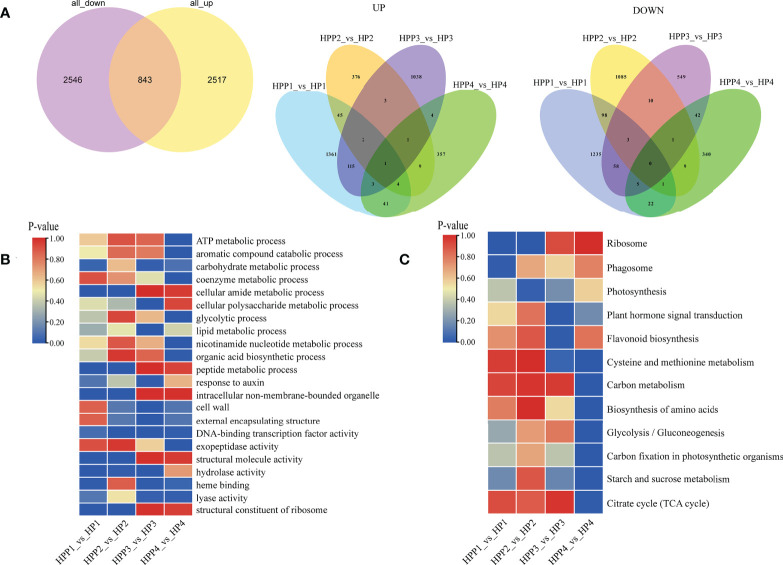
Analysis of functionally enriched differentially expressed genes (DEGs) between Harumi/Hongjv (HP) and Harumi/Ponkan/Hongjv (HPP) fruits. **(A)** Venn diagrams of all upregulated and downregulated genes between HP and HPP fruits. **(B)** Results of the Gene Ontology (GO) enrichment analysis of DEGs between HP and HPP fruits. **(C)** Results of the Kyoto Encyclopedia of Genes and Genomes (KEGG) pathway enrichment analysis of DEGs between HP and HPP fruits. HP1, HP2, HP3 and HP4 represent HP at 45, 90, 135, and 180 DAF, respectively; and HPP1, HPP2, HPP3 and HPP4 represent HPP at 45, 90, 135, and 180 DAF, respectively.

We compared the transcriptome profiles of the HP and HPP fruits at four developmental stages to identify DEGs during citrus fruit development (**Figure 4A** and **Supplementary Table 2**). For each pairwise comparison, the DEGs were filtered according to the following criteria: |log_2_ (fold-change)| in expression of >0 and an adjusted *P* < 0.05. We identified 5,906 DEGs between the HP and HPP groups, including 1572, 441,1167, and 420 upregulated genes in the HPP1, HPP2, HPP3, and HPP4 groups, respectively, and 1422, 1207, 668, and 420 downregulated genes in the HPP1, HPP2, HPP3, and HPP 4 groups, respectively (**Figure 4A**). Gene Ontology (GO) and KEGG analyses were conducted to functionally characterize the DEGs. The DEGs were commonly enriched in processes related to ribosomes, photosynthesis, plant hormone signal transduction, amino acid biosynthesis, glycolysis/gluconeogenesis, starch and sucrose metabolism, and the citrate cycle (**Figures 4B, C** and **Supplementary Table 2**).

The expression levels of genes related to carbohydrate metabolic processing were 1.3–10-fold higher in the HP1 group than in the HPP1 group. These upregulated genes included *PCK1* (LOC18037191), glucan endo-1,3-β-glucosidase 14 (LOC18034912), *TPS9* (LOC18044411), *KOR1* (ciclev10003884m), *BGL46* (LOC18052395), *TPS11* (LOC18054628), *XET 31* (LOC18034104), fructose-bisphosphate aldolase (*FBA*) genes (LOC18054367, LOC18034756, LOC18034756, LOC18054367), *CSLG3* (LOC18046063), alkaline/neutral invertase CINVs (LOC18042730, LOC18043168, LOC18031298), pyruvate kinase isozyme (*PKp3*, LOC18043314), inactive beta-amylase (*BMY3*, LOC18032416), and hexokinase (*HXK1*, LOC18035909). In contrast, the expression levels of several other genes were >2-fold higher in the HPP1 group, including genes encoding endoglucanases (LOC18048123, LOC18052685, LOC18033025), *PG* (LOC18031830, LOC18047316), *BGLU13* (LOC18044658), *XYL4* (LOC18041989), glucose-6-phosphate isomerase (*G6PI*, LOC18041337), and malate dehydrogenase (*MDH*, LOC18043642).

The expression levels of genes related to organonitrogen compound biosynthesis were 2–60-fold higher in the HP2 group than in the HPP2 group. These upregulated genes included *RPS30* (LOC18055660), *RPL34A* (LOC18048110), *SUI1* (LOC18055413), alkaline/neutral invertase (*CINV5*, LOC18031298), phosphoenolpyruvate carboxykinase (*PEPCK*, LOC18037191), cytochrome c (*CYTC2*, LOC18042136), several genes encoding transcription factors (TFs) in the ERF family (LOC18051602, LOC18041505, LOC18053449, LOC18047855, LOC18052831, LOC18031934, LOC18035271, LOC18047542, LOC18055444, LOC18033417, LOC18047855), and WRKYs (LOC18040890, LOC18038027, LOC18053740, LOC18040837, LOC18054113, LOC18053740). In contrast, the expression levels of several other genes were 1.2-fold higher in the HPP2 group, including genes encoding UTP-glucose-1-phosphate uridylyltransferase (*UGP1*, LOC18039774), 6-phosphofructokinase (*PFK4*, LOC18031386), sugar-phosphate translocator-related gene (LOC18047921), and pyruvate decarboxylase (*PDC*, LOC18039223).

The expression levels of several genes encoding xyloglucan endotransglucosylase/hydrolase proteins were >1.5–11-fold higher in the HPP3 group than in the HP3 group. These genes encode *XTRs* (LOC18035691, LOC18054158, LOC18054150, LOC18054151, LOC18041518), *XTHs* (LOC18031716, LOC18054144), *TCH4* (ciclev10010859m), phosphoenolpyruvate carboxylase (ATPPC4, LOC18043504), *FBA* (LOC18034756), pyruvate kinase (*PK*, LOC18032013), glyceraldehyde-3-phosphate dehydrogenase (*GAPA*, LOC18051549), NADH-quinone oxidoreductase (*NQR*, LOC18037766), auxin-responsive protein SAUR (LOC18044653, LOC18033610), two ERF-type TFs (LOC18046781, LOC18047939), WRKYs (LOC18050472, LOC18045437), phosphoenolpyruvate carboxykinase (*PEPCK*, LOC18037191), sucrose synthase (*SUS6*, LOC18038613), and cytochrome c (*CYTC2*, LOC18042136).

The expression levels genes encoding UDP-glycosyltransferase/sucrose synthase (SUS6, LOC18038613), serine carboxypeptidase-like (LOC18043305, LOC18039558, LOC18043732), pyruvate kinase (LOC18045215, LOC18050485), fructokinase-4 (*FRK4*, LOC18046804), and isocitrate dehydrogenase (*NAD-IDH*, LOC18037111) were >1.2-fold higher in the HP4 group than in the HPP4 group. In contrast, the expression levels of genes encoding pyruvate kinase (*PK*, LOC18046239), cytochrome P450 (*CYP82C4*, LOC18044013), *BR6OX1* (LOC18038016), *CYP71B35* (LOC18043324), *FAH1* (LOC18036838), *CYP71B37* (LOC18047711), and *CYP711A1* (LOC18054865) were 1.7–4.6-fold higher in the HPP4 group (**Supplementary Table 2**).

Ribosomes were affected by the interstocks (**Figure 4C** and **Supplementary Table 2**). Genes encoding several ribosomal proteins were repressed in the HP1 group, including ciclev10033291m, 50 S ribosomal protein L5 (LOC18046626), *RPL18* (LOC18048753), and *RPL36* (LOC18054512). However, the expression of genes encoding other ribosomal proteins were inhibited in the HPP2 group, including *RPS30C* (LOC18055660), *RPL34A*(LOC18048110), *ARS27A*(ciclev10017430m), *RS21*(LOC18041783), and a hypothetical protein (ciclev10003310m). Several genes encoding proteins important in plant hormone signaling and flavonoid biosynthesis were also downregulated by the interstocks in the HP3 group (**Figure 4C** and **Supplementary Table 2**), including *XTR6* (LOC18035691, LOC18054158, LOC18054151), *XTH22* (LOC18054150), *TCH4* (ciclev10010859m), *IAA29* (LOC18054871) encoding the xyloglucan endo-transglucosylase/hydrolase protein and cytochrome P450 gene *CYP75B1* (LOC18050323), flavonone isomerase gene *TT5* (LOC18043493), and flavanone-3-hydroxylase gene *F3H* (LOC18036490). Genes encoding proteins involved in carbon metabolism and amino acid biosynthesis were also downregulated by the interstocks in the HP4 group, including L-3-cyanoalanine synthase gene *CYSC1* (LOC18045998) and pyruvate kinase gene *PK* (LOC18046239).

To validate the RNA-seq results, several important DEGs were evaluated by quantitative reverse transcription-PCR (**Supplementary Figure 2A**). The expression patterns of the selected genes were consistent with the RNA-seq data.

### Differentially expressed endogenous metabolism-related genes at different developmental stages

**Figure 5:**
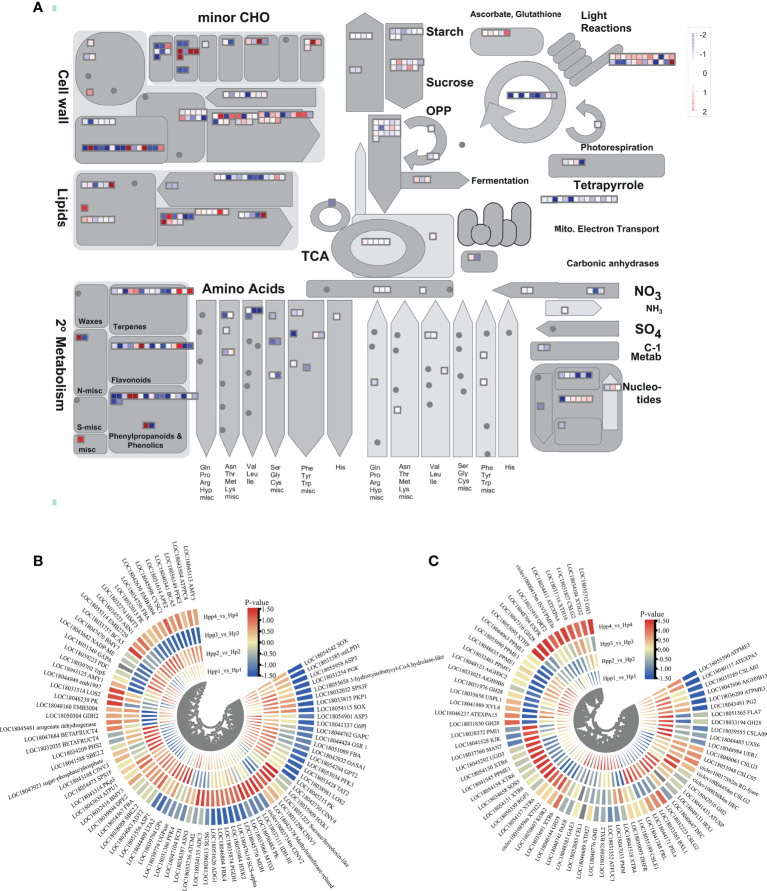
Differentially Expressed Endogenous Metabolism-Related Genes between Harumi/Hongjv (HP) and Harumi/Ponkan/Hongjv (HPP) fruits. **(A)** MapMan-based “metabolic-overview map” of differentially expressed genes (DEGs) (Harumi/Hongjv (HP) group vs Harumi/Ponkan/Hongjv (HPP) group) at four different developmental stages. **(B)** DEGs related to starch, sucrose, and tricarboxylic acid (TCA) metabolism during tangor fruit development and ripening. **(C)** DEGs related to cell wall metabolism during tangor fruit development and ripening. Heatmap of DEGs was drawn using the log2fold-change value obtained from the pairwise comparison of samples. Red and blue indicate upregulation and downregulation, respectively, in the former of the comparisons.

To understand changes in the transcript levels of metabolic pathways during HP and HPP fruit development and further investigate key DEGs, we obtained “metabolic-overview” profiles using MapMan (**Figure 5A**). The results showed that functional modules, such as lipids, cell wall metabolism, secondary metabolism, starch, sucrose, tricarboxylic acid (TCA) cycle, and amino acids, were regulated to varying degrees by interstocks. Here, we focused on DEGs related to sucrose and starch metabolism, the TCA cycle, and cell wall metabolism.

We analyzed the glycolysis pathway and associated DEGs (**Figure 5B**). We identified 84 DEGs between the HP and HPP groups associated with starch, sucrose metabolism, and the TCA cycle (**Supplementary Table 3**). Information pertaining to these genes is presented in a heat map (**Figures 5B–C**). Among the DEGs associated with starch and sucrose metabolism, the dihydrolipoyllysine-residue acetyltransferase gene *LTA2* (LOC18044409), dihydrolipoyl dehydrogenase gene *mtLPD1* (LOC18031585), and malate dehydrogenase gene *MDH* (LOC18045776) were upregulated in the HPP group at 45, 90, and 135 DAF and downregulated in the HPP group at 180 DAF. The isocitrate dehydrogenase gene *IDH-III* (LOC18037111), fructokinase gene *FRK4* (LOC18046804), alkaline/neutral invertase gene *CINV2* (CICLE_v10003734mg, LOC18042730), hexokinase gene *HXK1* (LOC18035909), and sucrase/ferredoxin-like gene (LOC18051223) were downregulated in the HPP group at 45, 90, and 180 DAF and upregulated in the HPP group at 135 DAF. The sucrose-phosphate synthase gene *SPS3F* (LOC18032032) was downregulated throughout all fruit-development stages in the HPP group. Two DEGs associated with the TCA cycle, *BMY3* (LOC18032416) and *DPE2* (LOC18039894), were downregulated throughout development and ripening in the HPP group, whereas the 1,4-alpha-glucan-branching enzyme gene *SBE2.2* (LOC18041588) and pullulanase gene *ATPU1* (LOC18043634) were downregulated in the HPP group at 45 DAF and upregulated in the HPP group at 90, 135, and 180 DAF.

DEGs related to cell wall metabolism are shown in **Figure 5C**. We identified 71 DEGs in all samples (**Supplementary Table 3**), of which 32, 34, 35, and 36 genes were differentially expressed at 45, 90, 135, and 180 DAF, respectively. Among the downregulated genes, *CSLG3* (LOC18046063) was downregulated during all stages. *INVI/PMEIs* (ciclev10000415m) and *GH3* (LOC18035312) were downregulated in the HPP group at 45, 90, and 135 DAF and upregulated at 180 DAF. *DHFR* (LOC18039093), *PAE8* (LOC18040742), and *GH9B6* (LOC18033025) were upregulated in the HPP group at 45 DAF and downregulated at 90, 135, and 180 DAF. Additionally, one gene, *GH28* (LOC18048144), was upregulated in the HPP group at all stages.

### Plant hormone-related and transcription-regulating degs at different developmental stages

**Figure 6:**
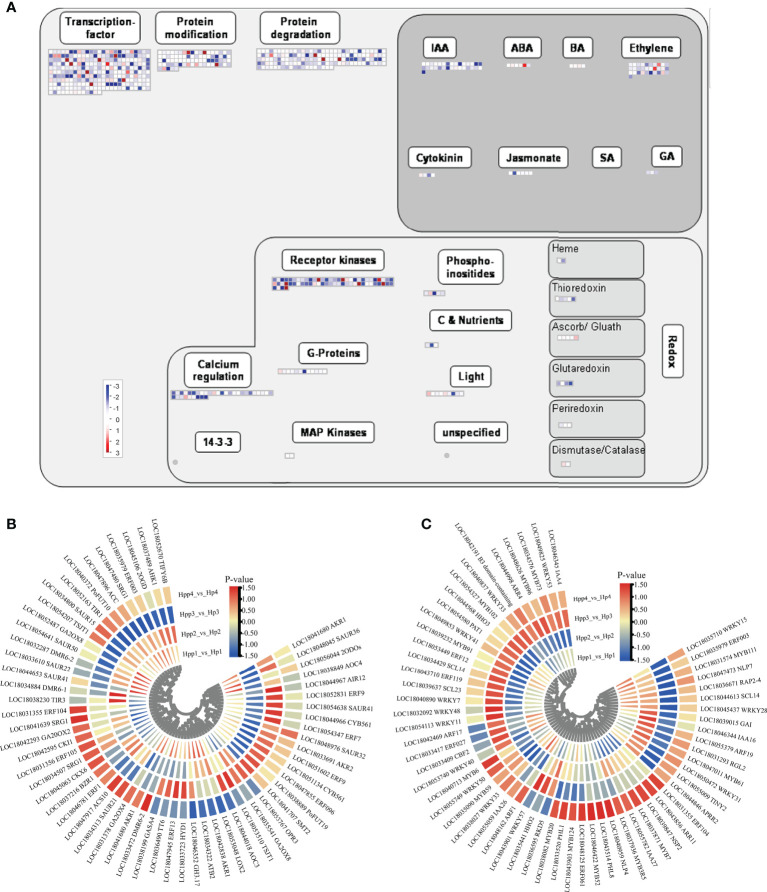
Plant Hormone-Related and Transcription-Regulating differentially expressed genes (DEGs) between Harumi/Hongjv (HP) and Harumi/Ponkan/Hongjv (HPP) fruits. **(A)** MapMan-based “regulatory-overview map” of differentially expressed genes (DEGs) (Harumi/Hongjv (HP) group vs Harumi/Ponkan/Hongjv (HPP) group). The small heat map in each section of the figure shows the DEGs mapped to the pathway, with a small square indicating a gene. The color of the square indicates the expression of that gene, with blue indicating downregulation and red indicating upregulation. **(B)** Phytohormone-related genes. **(C)** Transcription factor (TF)-related genes, where red and blue represent upregulated and downregulated TF genes, respectively.

We mapped the DEGs to a “regulatory-overview” graph using MapMan. For the hormone-biosynthesis pathway, 65 genes involved in growth hormone, abscisic acid, ethylene, gibberellin, and jasmonic acid synthesis were dysregulated by the interstocks during fruit development and ripening between the HP and HPP groups (**Figure 6A** and **Supplementary Table 3**). Most of these genes were related to the synthesis of ethylene (20 DEGs) and auxin (26 DEGs). The expression levels of genes encoding DMR6-like oxygenase (*DMR6*, LOC18033472) and ethylene-responsive TF *ERF104* (LOC18031355) were downregulated at 45 DAF and upregulated at 90, 135, and 180 DAF in the HPP group, whereas the gene encoding 2-oxoglutarate-dependent dioxygenase (*2ODOs*, LOC18056044) was upregulated at 45 DAF and downregulated at 90, 135, and 180 DAF in the HPP group. Only ethylene-responsive TF 1B (*ERF1*, LOC18046781) was downregulated in the HPP group at all stages. Among the six DEGs associated with gibberellic acid synthesis, a synthetic degradation gene (*GA20OX2*) was downregulated in the HPP group at all stages. The auxin-related genes *TIR1* (LOC18052163) and *TSJT1* (LOC18054207) and cytokinin-related gene *CKI1* (LOC18042595) were downregulated at 45, 90, and 135 DAF, whereas they were upregulated at 180 DAF in the HPP group (**Figure 6B**).

Over 260 DEGs encoding TFs were identified, including *AP2-EREBP* (APETALA2 and ethylene-responsive element-binding proteins), MYB, WRKY, bZIP, Aux/IAA, and C2H2 zinc finger proteins, as well as members of other major plant TF families (**Supplementary Table 3**). Genes encoding homeobox TF family (*HDG11*, LOC18050300), C2C2(Zn) DOF zinc finger family (*CDF2*, LOC18038335), MADS box TF family (*AGL22*, LOC18036998), MYB-related TFs (*MYB1R1*, LOC18040130; *RVE1*, LOC18039807; *HHO2*, LOC18035441), Kelch repeat-containing protein (*Krhp*, LOC18052245), and phosphatidylinositol N-acetylglucosaminyl transferase (*GPI1*, LOC18036233) were downregulated throughout development in the HPP group. Genes encoding Aux/IAA family (*IAA16*, LOC18046344; *IAA26*, LOC18055059), NIN-like bZIP-related family (*RKD5*, LOC18036595), nucleosome/chromatin assembly factor (*NFD4*, LOC18037272), MYB domain TF family (*MYB52*, LOC18046422), C2H2 zinc finger family (*IDD12*, LOC18049322), and growth-regulating factor (*GRF5*, LOC18041982) were upregulated throughout development in the HPP group (**Figure 6C**).

### Trend and venn analyses

**Figure 7:**
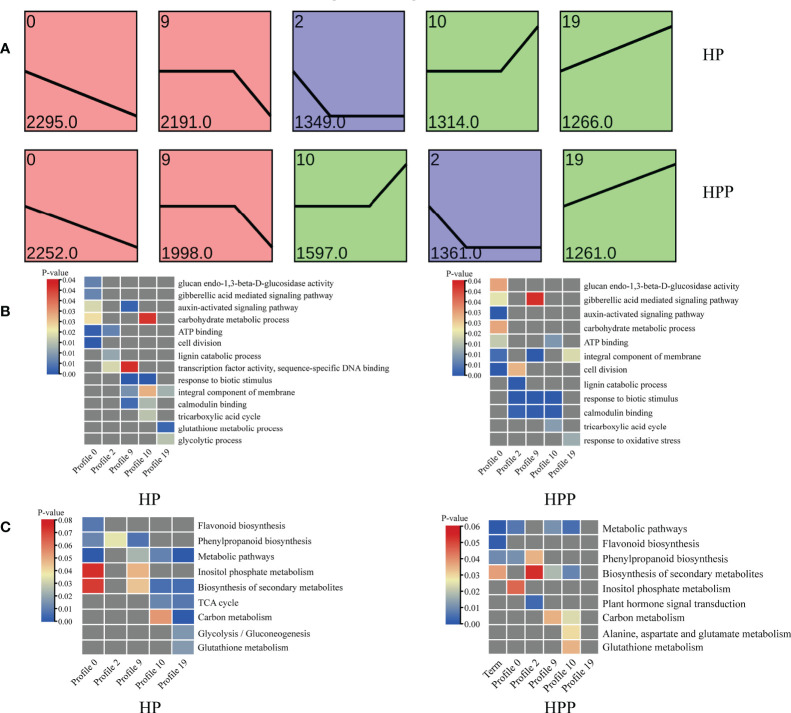
Gene-expression patterns and enrichment of Gene Ontology (GO) terms and Kyoto Encyclopedia of Genes and Genomes (KEGG) pathways at four different time-points in Harumi/Hongjv (HP) and Harumi/Ponkan/Hongjv (HPP) fruits. **(A)** Gene-expression patterns at three time-points in HP and HPP fruits, as predicted using STEM software. The number of genes and *P*-values for each pattern are indicated in each frame. **(B)** GO enrichment analysis of important processes in HP and HPP fruits. **(C)** KEGG pathway enrichment analysis of important processes in HP and HPP fruits. *P*-values indicate the significance of the most enriched GO and KEGG Slim terms. Significant and non-significant *P*-values are indicated in red and dark gray, respectively.

The DEGs at the four time-points in the HP and HPP groups were clustered into 10 profiles (**Supplementary Table 3**). Profiles 0, 2, 9, 10, and 19 were significantly overrepresented in the HP and HPP groups (**Figure 7A**). In the HPP group, genes related to carbon and energy metabolism (carbohydrate metabolic process, TCA cycle, carbohydrate binding, ATP binding, and electron carrier activity) were enriched in the above-mentioned profiles (**Figure 7B**). β-Galactosidase genes (*BGAL1*, LOC18037093; *BGAL17*, LOC18036862; *BGAL3*, LOC18036428; *BGAL5*, LOC18040789; *BGAL7*, LOC18055375; *BGAL8*, LOC18035117) and glucan endo-1,3-β-glucosidase genes (LOC18037713, LOC18050517; LOC18056170, LOC18036798, LOC18040076, LOC18052672, LOC18051455, LOC18051712, LOC18035509) were grouped into profile 0, whereas the MLO-like protein (*MLO*) gene was grouped into profiles 2, 9, and 10. Genes encoding ferredoxins (*FdC2*, LOC18033147, LOC18044482) were associated with profile 2. Genes encoding galacturonosyl transferases (*GAUT8*, LOC18049183, *GAUT10*, LOC18052790; *GAUT15*, LOC18045452) were grouped in profile 9. Genes encoding isocitrate dehydrogenase (*IDH1*, LOC18035427), 2-oxoglutarate dehydrogenase (*2-OGDH*, LOC18048455), phosphoenolpyruvate carboxylase (*ATPPC4*, LOC18043504), and succinate dehydrogenase (*SDH2*, LOC18037039) belonged to profile 10. Finally, genes encoding α,α-trehalose-phosphate synthase (*ATTPS7*, LOC18053226; *ATTPS9*, LOC18044411, *ATTPS6*, LOC18052900; *ATTPS11*, LOC18054628) were grouped in profile 19. Moreover, genes related to metabolic pathways, responses to oxidative stress, the biosynthesis of secondary metabolites, and plant hormone signal transduction were differentially expressed in the HP and HPP groups (**Figures 7B, C**).

**Figure 8:**
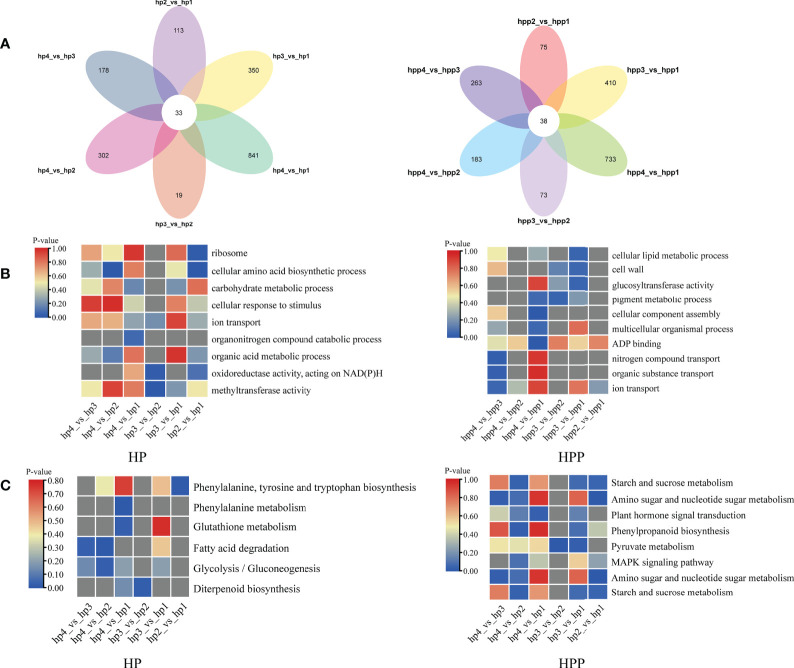
Venn analysis of differentially expressed genes (DEGs) over time in Harumi/Hongjv (HP) and Harumi/Ponkan/Hongjv (HPP) fruits. **(A)** Number of DEGs in the HP and HPP fruits. **(B)** Clusters of annotated Gene Ontology (GO) terms for DEGs in the HP and HPP fruits. **(C)** Kyoto Encyclopedia of Genes and Genomes (KEGG) pathway enrichment analysis of the DEGs in HP and HPP fruits. HP1, HP2, HP3 and HP4 represent HP at 45, 90, 135, and 180 DAF, respectively; and HPP1, HPP2, HPP3 and HPP4 represent HPP at 45, 90, 135, and 180 DAF, respectively. The significance of the most represented terms is indicated by the *P*-value. Significant and non-significant *P*-values are indicated in red and dark gray, respectively.

A Venn diagram was used to display the number of DEGs at the four stages in the HP (1836) and HPP (1775) groups (**Figure 8A** and **Supplementary Table 3**). Analysis of functional annotations revealed genes related to glucosyltransferase activity in the HPP group, including those encoding xyloglucosyl transferase (*XTH2*, LOC18031716; *XTH22*, LOC18054143), cellulose synthase-like protein (*CSLD4*, LOC18034163), and sucrose synthase (*SUS4*, LOC18054645). Additionally, genes associated with terms such as cellular lipid metabolic process, cell wall, multicellular organismal process, ADP binding, starch and sucrose metabolism, pyruvate metabolism, MAPK signaling pathway, and plant hormone signal transduction were enriched in the HPP group (**Figures 8B, C**). In the HP group, genes associated with terms for several activities and processes were enriched, including ribosome, cellular amino acid biosynthetic process, carbohydrate metabolic process, cellular response to stimulus, organic acid metabolic process, fatty acid degradation, and glycolysis/gluconeogenesis (**Figures 8B, C**).

### WGCNA and identification of critical modules

**Figure 9:**
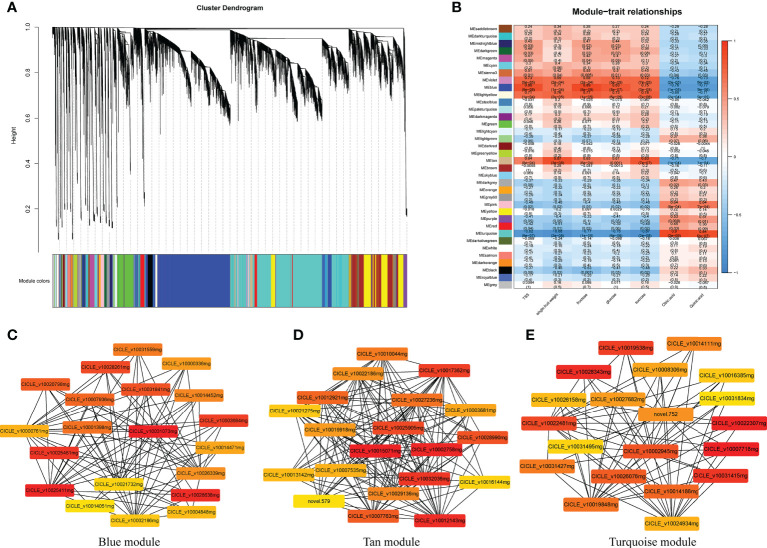
Weighted gene co-expression network analysis (WGCNA) of genes identified in Harumi/Hongjv (HP) and Harumi/Ponkan/Hongjv (HPP) fruits at all four developmental stages. **(A)** Thirty-six modules of co-expressed genes are shown in a hierarchical cluster tree. A major tree branch represents a module. The modules in the designated colors are shown in the lower panel. **(B)** Module–trait relationships. The remaining 36 modules are presented in the left panel. The module–trait correlation, ranging from -1 (blue) to 1 (red), is indicated by the color scale on the right. Each column presents the experimental traits, and their associations with each module are represented by a correlation coefficient and Pvalue in parentheses. **(C)** Gene network for the blue module, which positively correlated with TSS, fructose, and glucose. **(D)** Gene network for the tan module, which positively correlated with single-fruit weight and sucrose. **(E)** Gene network from the turquoise module, which positively correlated with citric acid and quinic acid.

Using the transcriptome data entered into the WGCNA module with FPKM values, 36 distinct gene modules were identified based on the co-expression patterns of individual genes. These gene modules are labeled in distinct colors and presented in a clustergram and network heatmap (**Figure 9A**). The TSS, single-fruit weight, fructose, glucose, sucrose, citric acid, and quinic acid levels at each developmental stage were used as phenotypic data to analyze the module–trait correlations. A sample dendrogram and trait heatmap were constructed to represent each phenotypic parameter at different developmental stages (**Figure 9B**). Of the 36 co-expressed gene networks, three modules (blue, tan, and turquoise shading) were closely connected with the seven above-mentioned traits (r > 0.8 and *P* ≤ 0.05; **Figure 9B**). The modules shown with blue shading were highly correlated with TSS, fructose, and glucose. Modules represented with tan shading were highly correlated with single-fruit weight and sucrose. Modules shown with turquoise shading were highly correlated with citric acid and quinic acid. Details regarding the genes in these modules are provided in **Supplementary Table 3**. Genes related to sugar, energy, hormones, and xylem metabolic processes were commonly identified in these modules.

Among the significantly correlated modules, gene pairs with the top 20 weight values for each trait (high connectivity) were used to construct networks (**Figures 9C–E** and **Supplementary Figure 2B**). In the blue-shaded module, the genes encoding histone deacetylase (*HD1*, LOC18034746), ATP-dependent zinc metalloprotease (*VAR2*, LOC18051570), and acyltransferase-like protein (LOC18052762) were closely related to other genes (**Figure 9C**). Additionally, genes encoding proteins related to sugar and energy, including malate dehydrogenase (*MDH*, LOC18043642), monogalactosyldiacylglycerol synthase (*MGD1*, LOC18054240), aldehyde dehydrogenase family 7 (*ALDH7B4*, LOC18036436), and sugar transporter ERD6-like 6 (LOC18036954), as well as cinnamoyl-CoA reductase (LOC18041277), coumarin 8-geranyltransferase (*HPT1*, LOC18042889), and chloride channel protein (*CLC-C*, LOC18043064) were also closely related to other genes (**Figure 9C**). Genes encoding cytochrome P450 reductase (*ATR2*, LOC18055638), the sugar transporter ERD6-like 7 (LOC18046354), l-ascorbate peroxidase 3 (*APX3*, LOC18035392), tryptophan aminotransferase-related protein 3 (LOC18043155), uridine nucleosidase 1 (*URH1*, LOC18038155), tryptophan synthase α chain (*TSA1*, LOC18046349), and NADH dehydrogenase (*FRO1*, LOC18042718) were identified as hub genes in the tan-shaded module (**Figure 9B**). The turquoise-shaded module consisted of eight hub genes, including the FAD-binding PCMH-type domain-containing protein (*AAO3*, CICLE_v10027682mg), protein aspartic protease in guard cell 1 (*ASPG1*, LOC18048745), taxadien-5-α-ol O-acetyltransferase (LOC18044180), probable β-1,4-xylosyltransferase (*IRX9H*, LOC18055329), auxin-responsive protein (*IAA26*, LOC18035868), a TF (*MYB16*, LOC18048141), serine carboxypeptidase-like 35 (*scpl35*, LOC18044336), and type-I inositol polyphosphate 5-phosphatase 4 (*INPP5A*, LOC18047168).

## Discussion

Fruit development and maturation are complex biological processes regulated by various environmental, hormonal, and genetic factors (Ye et al., 2021). Rootstocks greatly affect plant growth, productivity, and fruit quality (Hu et al., 2022). Regulatory pathways related to biological sugars and acids serve as vital metabolic constituents during fruit development and maturation. In this study, we explored the complex genetic factors affecting variations in the quality of citrus fruit.

### Sugars and organic acids in citrus fruit affected by interstocks

The stages of citrus fruit development are cell-division, expansion, and ripening (Tadeo et al., 2008). Fruit development is typically accompanied by sugar accumulation and organic acid degradation (Batista-Silva et al., 2018; Bertrand et al., 2018). Three important carbohydrates (fructose, glucose, and sucrose) are found in a 1:1:2 ratio in whole fruits (Kelebek and Selli, 2011). Our data showed that the levels of sucrose, fructose, and glucose varied during different developmental stages, suggesting that sugar metabolism in fruits mainly depends on developmental processes. The sucrose, fructose, and glucose contents were low in the HP and HPP groups during the initial fruit-development stages. Toward maturity, these compounds accumulated at high levels, and a significant difference was observed between the HP and HPP groups, particularly in terms of the sucrose content, which substantially increased near maturity. Taken together, these data indicate that the sucrose content was higher than the fructose and glucose contents, which is similar to previous findings (Kelebek and Selli, 2011).

In contrast to the changes in sugar metabolism, organic acids typically accumulate during early stages of fruit development and decrease during fruit ripening and storage (Zhu et al., 2017). This decrease in organic acids at later stages results from the enhancement of sugar synthesis and secondary metabolic pathways toward ripening (Lombardo et al., 2011). We observed similar trends in these acids: early accumulation of citrate and quinic acid was observed; as maturity process continued, the citrate and quinic acid contents decreased. Citric acids were the most abundant organic acids found in both climacteric and non-climacteric ripe fruits (Batista-Silva et al., 2018). Physiological changes during citrus fruit development can lead to substantial changes in the single-fruit weight, TSS, and acid content, thereby affecting the overall quality of citrus fruits. Therefore, it is important to identify the key genetic factors regulated by interstocks, including gene networks and major contributors, by controlling the variations in these compounds, particularly sugars and organic acids.

It is generally thought that the carbohydrate-distribution pattern in a plant is related to the relative competitive ability of various sink regions within the whole plant (Simkhada et al., 2007). Because of the specific location of interstocks, some researchers proposed that changes in the structure of transfusion tissues of graft unions (interstock–scion and interstock–rootstock unions) and phloem sieve-tube elements hinder downward transport of photosynthates, and that this blockage affects the vigor of the aerial parts of the tree (Zhou et al., 2015). A previous study of apples suggested that GM256 interstocks can induce growth reduction, branching, and early fruiting and increase fruit productivity and quality (Karlıdağ et al., 2014). Wang et al. (2003) found that differences in the photosynthate distributions of the two graft combinations between aboveground organs and underground organs do not necessarily result from blockage of the photoassimilate flow in interstocks, and that the differences may be caused by changes in the sink strength. In this study, interstocks improved the TSS of citrus fruits of HPP. Trees with interstocks showed higher sugar contents and lower acid contents compared to in non-interstocked trees. The differences in sugar and acid contents between the two combinations were caused by the interstocks, as they were grown under the same conditions and using the same materials, scions, and rootstocks.

### DEGs between HP and HPP citrus trees in plant hormone signal transduction, carbohydrate metabolism, and the TCA promoted fruit ripening by interstocks

Phytohormones regulate fruit maturation and ripening *via* synergistic and antagonistic interactions (Zhao et al., 2021). Auxin and ethylene pathways cooperatively regulate numerous developmental processes in plants (An et al., 2020). Huang et al.(2020) observed that exogenous cytokinins treatment can increase the density and the length of root. Corot et al. (2017) found that abscisic acid and cytokinins antagonistically regulate bud outgrowth. Studies on apples showed that dwarfing rootstocks accelerate fruiting precocity, possibly *via* partitioning of carbohydrates and hormones into fruits (Avery, 2010). Comparison of HP and HPP citrus trees revealed DEGs related to plant hormone signal transduction, starch and sucrose metabolism, amino acid biosynthesis, and the TCA cycle (**Figures 2B, C** and **Supplementary Table 2**). The expression levels of ethylene-responsive TF 1B (*ERF1*, LOC18046781), synthetic degradation gene (*GA20OX2)*, auxin-related genes *TIR1* (LOC18052163) and *TSJT1* (LOC18054207), and cytokinin-related gene *CKI1* (LOC18042595) were downregulated in the HPP group. In many fruits, decreases in auxin levels occur during the initial stages of ripening (Tobaruela et al., 2021). In *Arabidopsis thaliana*, inhibiting *GA20OX2* expression can prevent precocious flowering (Susila et al., 2020). These findings suggest that interstocks promote citrus precocity through the plant hormone signaling pathway, in which *ERF1* and *GA20OX2* may be key factors, although this possibility should be verified experimentally.

We observed that sucrose-, TCA -, and cell wall-related genes (e.g., *CINV2*, *FRK4*, *HXK1*, *SPS3F*, *BMY3*, *DPE2*, *CSLG3*, and *INVI/PMEIs*) were downregulated in HPP citrus samples (**Supplementary Table 2**). Furthermore, 8, 4, 14, and 6 genes were exclusively expressed in HPP flesh harvested after 45, 90, 135, and 180 days, respectively (**Supplementary Table 2**). Interestingly, these DEGs were also related to the harvest time. Functional exploration of these genes suggested their roles in multiple pathways. For example, genes expressed in the HPP1 flesh harvested after 45 days included the SRF-type TF (Lai et al., 2021) and AAA-ATPase. Genes expressed in the HPP2 flesh harvested after 90 days were related to sphingolipid C9-methyltransferase 1, CBF/NF-Y (Dolfini et al., 2012), and BCS1-like protein. HPP3 flesh harvested after 135 days exclusively expressed aluminum-activated malate, protein tyrosine kinase, aromatic-rich glycoprotein, polygalacturonase, monothiol glutaredoxin S1, and glutaredoxin. HPP4 flesh harvested after 180 days exclusively expressed SKP1-like protein, protease inhibitor, seed storage, LTP family members, GDSL-like lipase, and acylhydrolase. A total of 19, 14, 10, and 4 genes was exclusively expressed in HP flesh harvested after 45, 90, 135, and 180 days, respectively. The genes expressed in HP1 flesh were related to cytochrome P450 (Mak and Denisov, 2017), the TF *bHLH96* (Wang et al., 2019), glutaredoxin, UDP-glycosyltransferase 13, C-glycosyltransferase, and the transferase family. Genes expressed in HP2 flesh were related to the *Sec1* family (Matthias et al., 2018), troponin reductase homolog, Ctr copper transporter family, and germin-like protein subfamily 1 member 15. HP3 flesh showed exclusive expression of the auxin-responsive protein SAUR19 (Kathleen and Farquharson, 2014), zinc-binding dehydrogenase, non-specific lipid-transfer protein 3, vinorine synthase, and transferase family. HP4 flesh exclusively expressed *trans*-resveratrol di-O-methyltransferase and UDP-glucuronate 4-epimerase 5 (Iacovino et al., 2020). These exclusively expressed genes may have affected the observed phenotypes.

TFs play important roles in fruit development. Our transcriptome analysis revealed that 30 AP2/EREBP, 22 MYB, 18 bZiP, 13 WRKY, and 13 bHLH family TFs were differentially expressed between the HP and HPP groups at four fruit-development stages (**Supplementary Table 3**). Some predicted target genes of these TFs may be functionally related to fruit ripening, such as *MYB* (Fu et al., 2020), *Aux/IAA* (Su et al., 2015), and *GRFs* (Cao et al., 2016). Our results showed that several TFs (e.g., *IAA26*, *IAA16*, *MYB52*, and *GRF5*) were upregulated in HPP citrus samples (**Supplementary Table 2**). The effects of interstocks on citrus fruit development and quality were explored at four different time-points. The results of profile analysis indicated that genes related to carbohydrate metabolism, the TCA cycle, cell wall metabolism, starch, sucrose, and pyruvate metabolism were enriched in the HPP group (**Figures 5B, C**). Moreover, the contents of most carbohydrates analyzed in the HPP group changed substantially over time. Venn analysis indicated that hormone metabolism was enriched in HPP trees compared to in HP trees (**Figures 6B, C**).

### Hub genes in the wgcna module control sugar, hormone, and xylem metabolism, along with cell growth

The WGCNA results indicated that processes related to sugar, energy, hormone, carbohydrate, and cell wall metabolism correlated significantly with the target traits, which is consistent with the results of DEG analysis (**Figure 9**). Complex biochemical pathways, such as sugar and organic acid synthesis, are regulated by multigene responses and cannot be explained by individual genes (Umer et al., 2020). The fruit quality of HPP and HP trees was closely related to cytokinins, ethylene, gibberellins, and indoleacetic acid. Previous data showed that grafting can affect the fruit quality through different biological processes, such as by inducing different genes (Aslam et al., 2020) and causing changes in enzyme activities (Sucu et al., 2018). We found that growth-regulating factor *GRF10*, sugar transporter ERD6-like, glycosyl transferase gene *IRX9H*, auxin gene *IAA26*, and malate dehydrogenase gene *MDH* were highly correlated with other genes in significant modules, indicating that the interstocks affected the crosstalk between hormones and genes related to glycoacid metabolism. *MYB16* was also identified as a hub gene. Considering the positive effects of interstocks on citrus fruit quality, interstock mediation may promote the early ripening of fruits. These hub genes should be further examined to determine their roles in interstock-mediated citrus fruit ripening.

## Conclusion

**Figure 10:**
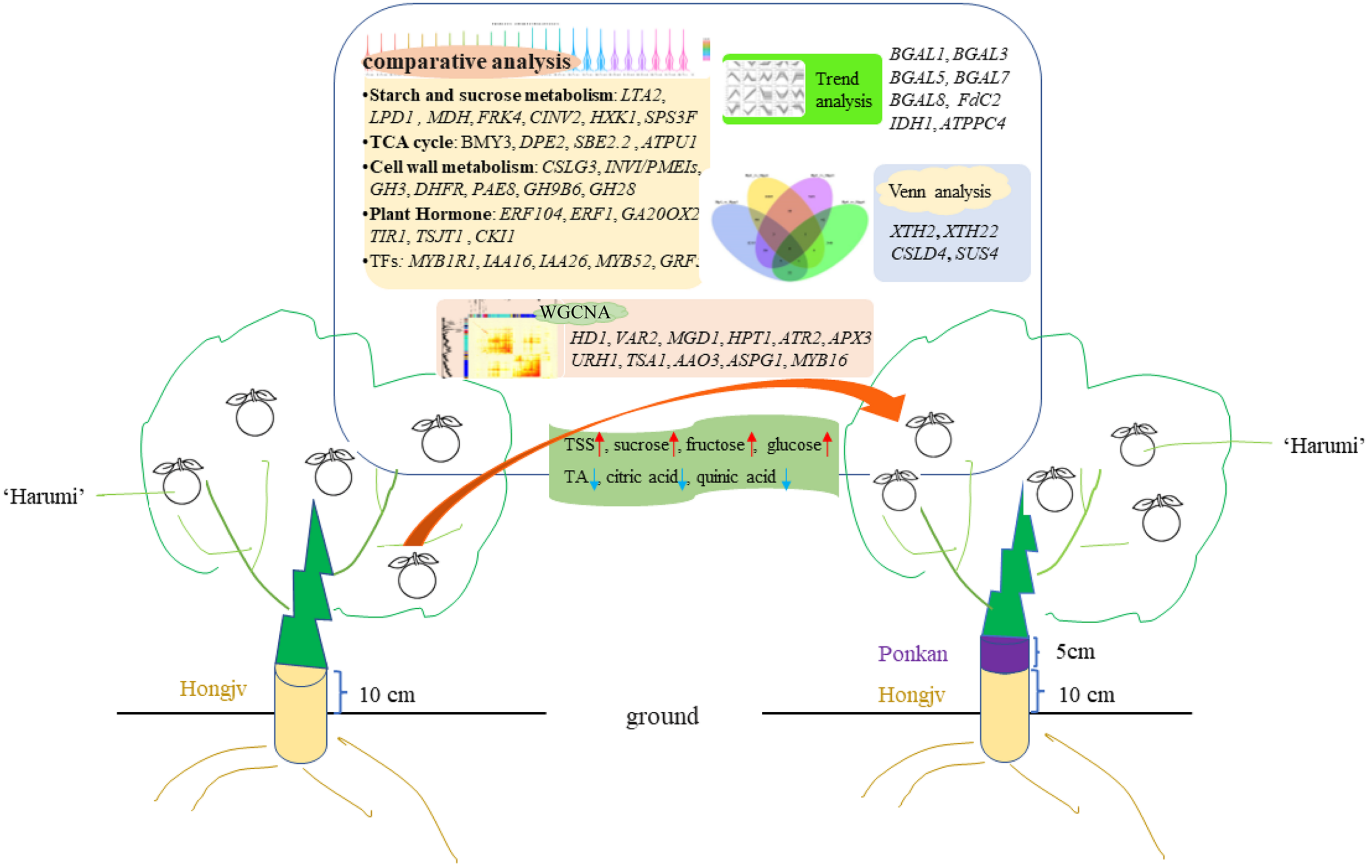
Proposed model for the interstock-mediated Harumi Tangor quality. A comparative analysis revealed that starch and sucrose metabolism, TCA cycle, cell wall metabolism, and Plant hormone are affected during fruit riping. Up arrows (red) represent raising, and down arrows (blue) represent lowering. Crucial genes based on the trend analyses, Venn analyses, and a WGCNA are indicated.

We determined transcriptome profiles to investigate the gene networks controlling the regulation of sugars and organic acids in citrus based on co-expression patterns. Specifically, interstocks may induce early fruiting and increase fruit quality by upregulating sucrose, fructose, and glucose contents, as well as by decreasing organic acid contents. The phytohormone signal is activated by alterations in the expression levels of *ERF1*, *GA20OX2*, *CKI1*, and *TIR1*. Genes related to sugar metabolism (i.e., starch, glucose, sucrose, fructose, and TCA) and energy metabolism (e.g., *CINV2*, *FRK4*, *HXK1*, *SPS3F*, *BMY3*, *DPE2*, *CSLG3*, and *INVI/PMEIs*) or those encoding TFs (e.g., *MYB52*, and *GRF5*) were strongly affected by interstocks during fruit ripening. WGCNA revealed that genes related to the growth-regulating factor *GRF10*, sugar transporter ERD6-like, glycosyl transferase gene *IRX9H*, auxin gene *IAA26*, malate dehydrogenase gene *MDH*, and TF *MYB16* were hub genes between the HP and HPP groups (**Figure 10**). These factors commonly regulate citrus fruit quality and ripening.

Our results suggest that interstocks can lead to fluctuations in auxin, ethylene, and other plant hormones, as well as alter the cell wall, sugar, and energy metabolism during fruit development, which in turn regulate citrus fruit quality. However, how these elements actively regulate the quality of citrus fruit remains unclear. These phenomena in perennial fruit trees are complex and require clarification in future studies. Our data will enable further studies aimed at characterizing the molecular mechanisms regulating interstock-mediated growth.

## Data availability statement

The datasets presented in this study can be found in online repositories. The names of the repository/repositories and accession number(s) can be found in the article/**Supplementary Material**.

## Author contributions

Project and experiment design, ZW and LL; experiment execution, LL, YL, XB, BX, XW, HD, and MZ; data analysis, ZJ, ZH and GS; writing, LL; review, BX and HD; project management, GS and ZW. All authors contributed to the article and approved the submitted version.

## Funding

This research was funded by the National Key R&D Program of the Ministry of Science and Technology project (2021YFD1600800) and Sichuan Science and Technology Department Projects (2021YJ0486).

## Acknowledgments

We thank all editors and reviewers for their critical reading of the manuscriptand their suggestions.

## Conflict of interest

The authors declare that the research was conducted in the absence of any commercial or financial relationships that could be construed as a potential conflict of interest.

## Publisher’s note

All claims expressed in this article are solely those of the authors and do not necessarily represent those of their affiliated organizations, or those of the publisher, the editors and the reviewers. Any product that may be evaluated in this article, or claim that may be made by its manufacturer, is not guaranteed or endorsed by the publisher.

## Abbreviations

DEG, differentially expressed gene; FPKM, fragments per kilobase of transcript per million mapped reads; GO, Gene Ontology; HPLC, high-performance liquid chromatography; KEGG, Kyoto Encyclopedia of Genes and Genomes; PCR, polymerase chain reaction; RNA-seq, RNA sequencing; TCA, tricarboxylic acid cycle; TF, transcription factor; TSS, total soluble solids; WGCNA, weighted gene co-expression network analysis.
