# Supplementary material for: Transcriptome analysis of Harumi tangor fruits: Insights into interstock-mediated fruit quality
Source: Front Plant Sci. 2022 Oct 13;13:995913. doi: 10.3389/fpls.2022.995913 (PMC9608513; doi:10.3389/fpls.2022.995913)

**Supplementary Figure 1.** A) Distribution of sample gene expression and B) Pearson correlation coefficients in ‘Harumi’ Tangor fruit peels in different treatment comparisons. HP1, HP2, HP3 and HP4 represent HP samples at 45, 90, 135 and 180 days after flowering. HPP1, HPP2, HPP3 and HPP4 represent HPP samples at 45, 90, 135 and 180 days after flowering. The numbers 1, 2, and 3 with each treatment represent the three replicates.


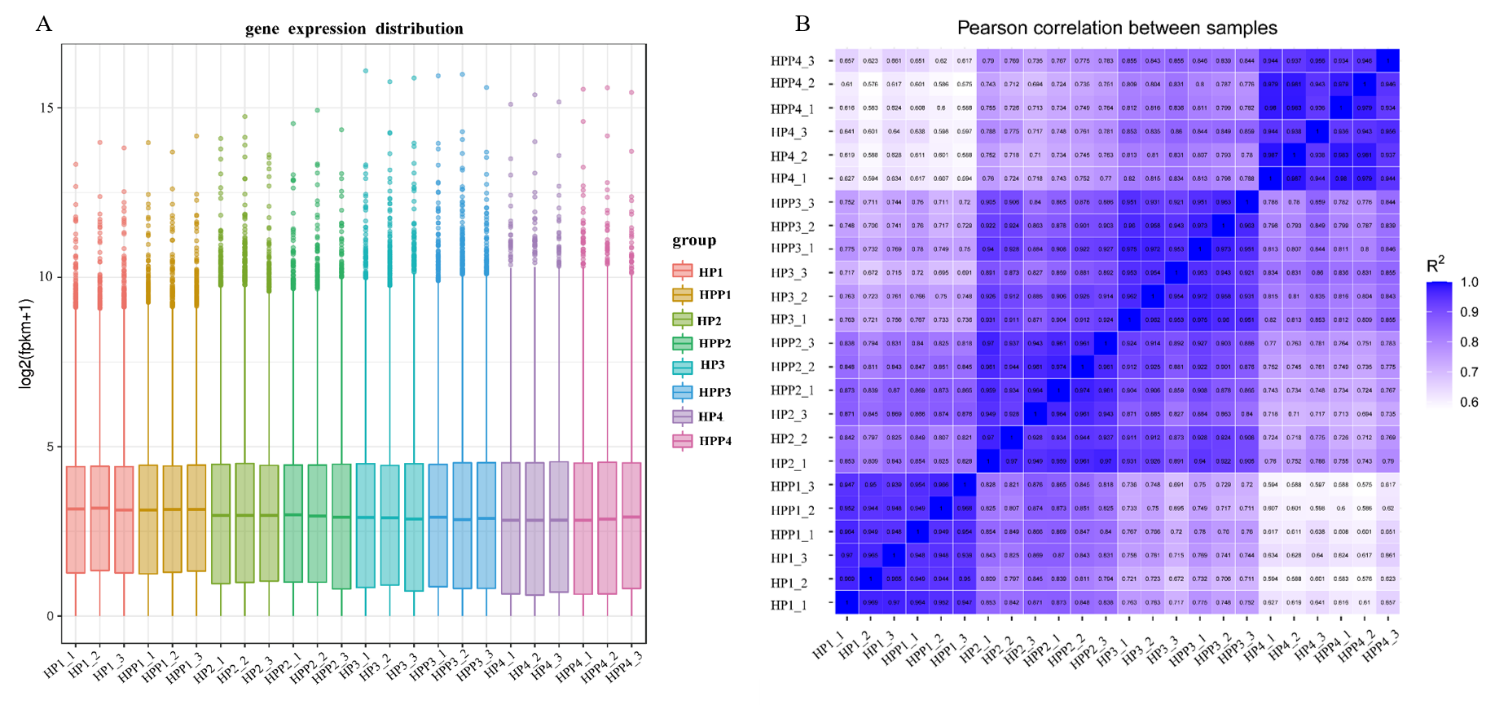


**Supplementary Figure 2.** Quantitative RT-PCR analysis. (A) DEGS based on the results of comparative analysis; (B) DEGS based on the results of WGCNA analysis.


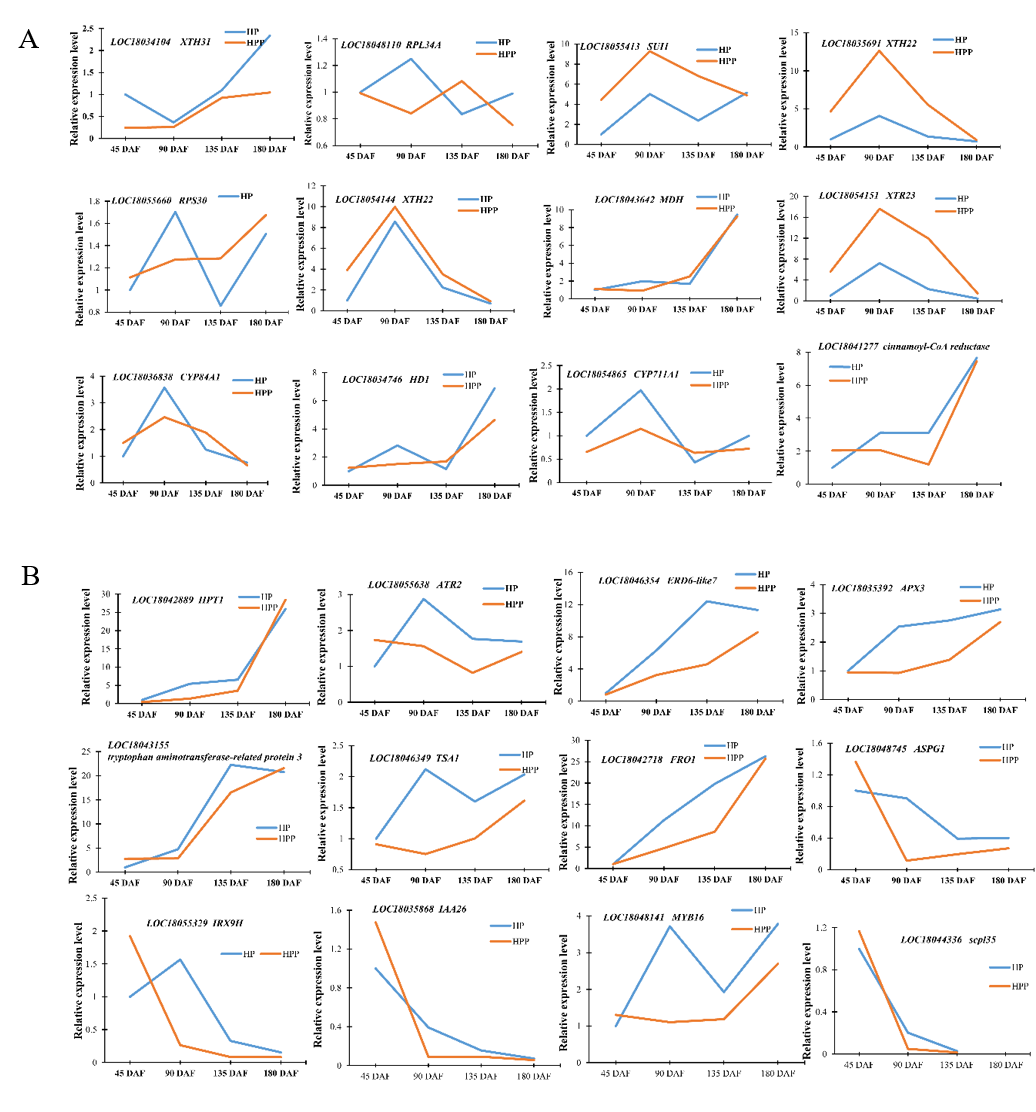

Supplement: Supplementary file 2 [file DataSheet_2.docx]
